# Case of Inversion of the Uterus, Treated Successfully by Elastic Pressure

**Published:** 1870-08

**Authors:** W. H. Byford

**Affiliations:** Prof. of Obstetrics in Chicago Med. College


ARTICLE XIX.

### CASE OF INVERSION OF THE UTERUS, TREATED SUCCESSFULLY BY ELASTIC PRESSURE.

BY W. H. BYFORD, A.M., M.D., Prof. of Obstetrics in Chicago Med. College.

Mrs. H. N. H., aged 22 years, was confined with her third child, at 8.30 o'clock A.M., on the 9th of March, 1870. The labor was about four hours duration. The placenta was expelled spontaneously, in about half an hour after the child. Very little blood accompanied the placenta. As usual, the hand was placed over the hypogastric region, in order to ascertain the condition of the uterus. The organ was large and soft, and in 15 or 20 minutes after the delivery of the placenta, a copious hemorrhage commenced, but was easily controlled by grasping the uterus, through the abdominal wall. The contractions resulting from this treatment were prompt and obvious. I remained with the patient something like an hour and a-half, when the hemorrhage was completely controlled and the uterus well contracted. At 12 o'clock, I was summoned by a messenger from the patient, with the information that she was flooding copiously. When I arrived, I found her prostrated very decidedly by loss of blood, and still bleeding. The uterus was large, reaching above the umbilicus, and very soft. Pressure and grasping caused it again to contract and throw out a large quantity of coagula and fluid blood. Fluid extract of ergot was administered as soon as it could be procured, cold applied to the urethra, and continued pressure kept up. The hemorrhage soon entirely ceased, the uterus contracted well, and the contractions were painful more than ordinarily. The hemorrhage did not return, but the patient had already lost so much blood, that she was very greatly prostrated. The pulse was weak and rapid, perhaps 120 strokes to the minute. Upon the slightest exertion, a sense of fainting was produced. She remained prostrated, so as to require stimulation and concentrated nourishment for 36 hours. From this time gradually rallied, and in 20 days was able to sit up some, while the pulse had regained its volume and usual frequency. From this time, she improved quite rapidly, and, in four weeks, was riding and even walking about the streets some, with no special discomfort. The lochia was still somewhat copious, but colorless, and "watery," as she expressed. On the 25th, the lochia was so abundant that I was led to make an examination. I found the cervix large and soft, but otherwise there was nothing unusual. The uterus could still be felt above the pubis. On the 28th, I left the city for two months, and, consequently, did not know anything of her condition personally, until I returned on the 5th of June, and, consequently, rely upon her account of the progress of things.

She informed me that she continued to improve, and, as above stated, in about four weeks, was riding out, and walking about the house, and on the street, without inconvenience, except such as arose from debility, and that was not very considerable. On the 14th or 15th of April, while sitting on the vessel vomating, she experienced slight tenesmus, and felt something pass out between the labia. Placing her hand upon the parts, she discovered a protrusion. She passed it back and sent for a physician. It was through the examination made at that time that she was found to have an inverted uterus. She was treated with sitz-baths and astringent injections into her vagina, for three or four weeks, when she was pronounced cured, and discharged. When I returned home, I saw her on the 5th of June, and found the uterus inverted. It was about the ordinary size of the unimpregnated organ, and inverted all but the vaginal portion of the cervix. The density of the anterior walls and cervix seemed rather less than natural. Since she first discovered the protruding organ, the patient had been subject to small losses of blood, and between these, a serous or watery, and sometimes mucus discharge from the vagina. Her general condition was tolerably good, although she had not entirely recovered from the debility caused by the copious hemorrhage.

After lifting up the uterus as much as I could with two fingers, I placed an elastic bag under it, so as to make pressure upward and slightly forward, in the direction of its axis, and filled with tepid water, until the patient complained of severe, inconvenient pain, from distention. She was directed to remain in bed, and the instrument was left in place until the next day. The water was then allowed to flow out and the parts examined. I could discover no change in the condition of the uterus. The bag was again filled and the distention made as great as the patient could well bear. Upon emptying it again, on the third day, relaxation of the cervix was quite evident, and I felt encouraged to expect success. On the fourth day, the relaxation was so decided, that the fundus could be almost passed up into the os with the fingers. On the fifth day, when the water was again allowed to flow off, the uterus was found to have returned to its natural position. The patient was directed to remain in bed still, and the instrument removed from the vagina. On the sixth day, I passed my probe through the os uteri, two and a-quarter inches, showing that the fundus was thoroughly restored to its shape and position. The patient remained in bed for two days more, and then gradually resumed her exercise until, on the 22d of June, she attended the wedding of one of her friends. This case is remarkable from the manner of occurrence and for the ease with which the reduction of the inversion was effected. I think the last fact shows a very pliable condition of the organ, and, to some extent, explains the method of the accident. With reference to this, it is quite evident that the time of the completion of the inversion must have been the time of its discovery; for if the process of involution, at that late period, was so imperfect, as that the uterus was large enough to pass, in considerable size, beyond the labia, it is reasonable to suppose, that had it been completely inverted before, it would have made its appearance before and hence attracted the attention of the patient. When I first saw her upon my return, the involution appeared to be complete, and the organ was distant from the vaginal orifice. It certainly was not inverted at the end of three weeks from labor, and it was unquestionably larger at that time than usual. I do not believe that there was indentation, or anything indicating the commencement of the process of inversion, on the 25th of March, when last I saw her before the accident. I am glad to have an opportunity of recording this case, as showing the great efficiency of elastic pressure, in the correction of the condition. It was very evident, from the daily observation of the case, that the reduction was effected, not by indenting the fundus, but by the relaxation of the cervix, and the passage of the whole body and fundus through it, and that the fundus was the last part to be rectified. The last labor of the patient, previous to this, was attended by very copious hemorrhage and almost as great prostration as stated in this; and I think it would not be unreasonable to infer, that my patient only represents a class of patients, in whom the habitually flaccid condition of the uterus predisposes them to the accident.

This case, in many respects, is quite similar to one that occurred in the practice of Dr. A. Fisher, of this city, an experienced, intelligent, and careful practitioner. It is recorded, in the October number of the *Chicago Medical Journal*, for the year 1858. Dr. Fisher discovered the inversion on the 18th of May, 38 days after delivery. No symptoms, except slight hemorrhage, indicated anything wrong. The Doctor says: "I made a digital examination, and, to my utter astonishment, found the uterus completely inverted, the fundus resting on the peritoneum." Dr. N. S. Davis, who was called to see the patient, in consultation with Dr. Fisher, regarded it as a case of spontaneous inversion, and said, in reference to it, in an editorial, in the same number of the *Journal*, "I questioned both the patient and her mother carefully and minutely, in regard to what transpired at the time of confinement, and I elicited no facts differing from the statements given by Dr. Fisher."

